# Responsiveness of various reservoir species to oral rabies vaccination correlates with differences in vaccine uptake of mucosa associated lymphoid tissues

**DOI:** 10.1038/s41598-020-59719-4

**Published:** 2020-02-19

**Authors:** Verena te Kamp, Conrad M. Freuling, Ad Vos, Peter Schuster, Christian Kaiser, Steffen Ortmann, Antje Kretzschmar, Sabine Nemitz, Elisa Eggerbauer, Reiner Ulrich, Jan Schinköthe, Tobias Nolden, Thomas Müller, Stefan Finke

**Affiliations:** 1 Institute of Molecular Virology and Cell Biology, Friedrich-Loeffler-Institut, WHO Collaborating Centre for Rabies Surveillance and Research, OIE Reference Laboratory for Rabies, Greifswald-Insel Riems, Germany; 2 Ceva Innovation Center GmbH, 06861 Dessau-Rosslau, Germany; 3 Department of Experimental Animal Facilities and Biorisk Management, Friedrich-Loeffler-Institut, Greifswald-Insel Riems, Germany; 4 Present Address: Thescon GmbH, 48653 Coesfeld, Germany; 5 Present Address: BioNTech IMFS GmbH, 55743 Idar-Oberstein, Germany; 6 Present Address: Thüringer Landesamt für Verbraucherschutz, 99947 Bad Langensalza, Germany; 7 Present Address: Institute of Veterinary Pathology, Faculty of Veterinary Medicine, Leipzig University, 04103 Leipzig, Germany; 8 Present Address: ViraTherapeutics GmbH, 6020 Innsbruck, Austria

**Keywords:** Live attenuated vaccines, Virus-host interactions

## Abstract

Oral rabies vaccination (ORV) is highly effective in foxes and raccoon dogs, whereas for unknown reasons the efficacy of ORV in other reservoir species is less pronounced. To investigate possible variations in species-specific cell tropism and local replication of vaccine virus, different reservoir species including foxes, raccoon dogs, raccoons, mongooses, dogs and skunks were orally immunised with a highly attenuated, high-titred GFP-expressing rabies virus (RABV). Immunofluorescence and RT-qPCR screenings revealed clear differences among species suggesting host specific limitations to ORV. While for responsive species the palatine tonsils (*tonsilla palatina*) were identified as a main site of virus replication, less virus dissemination was observed in the tonsils of rather refractory species. While our comparison of vaccine virus tropism emphasizes the important role that the tonsilla palatina plays in eliciting an immune response to ORV, our data also indicate that other lymphoid tissues may have a more important role than originally anticipated. Overall, these data support a model in which the susceptibility to oral live RABV vaccine infection of lymphatic tissue is a major determinant in vaccination efficacy. The present results may help to direct future research for improving vaccine uptake and efficacy of oral rabies vaccines under field conditions.

## Introduction

Rabies is a primary example of how oral vaccination aids to the control and elimination of an infectious disease with zoonotic or economic relevance, particularly with regards to wildlife. In principle, vaccination of wildlife reservoir species should result in a herd immunity above a threshold where the transmission cycle of the disease ceases to persist^1,2^. While wildlife rabies in Eurasia is mainly associated with foxes and raccoon dogs^3^, in the Americas, raccoons and skunks both serve as major reservoir species and potent transmitter of the disease to domestic animals^4^. Another important reservoir in the Caribbean and Southern Africa are mongooses^5^.

The domestic dog represents the main reservoir and source of infection for humans, particularly in developing countries in Africa and Asia^6^. Oral vaccination of free-roaming dogs is considered an important complementary tool to increase herd immunity and thus the likelihood of disease elimination^7^.

Field effectiveness of oral vaccination campaigns is influenced by factors such as the composition of vaccine-loaded baits and a strategy of bait distribution. Another important component is the use of an efficacious and safe oral rabies vaccine. While oral rabies vaccination campaigns using either attenuated or recombinant vaccines have been successful in red foxes (*Vulpes vulpes*), gray foxes (*Urocyon cinereoargenteus*) and coyotes (*Canis latrans*) in North America^8–10^ and in red foxes and raccoon dogs (*Nyctereutes procyonoides*) in Europe^3,11^, there seems to be inefficient or variable efficacy of oral immunisation in some other target species^12^. In fact, there is limited success in the oral vaccination of raccoons (*Procyon lotor*) and striped skunks (*Mephitis mephitis*) as shown experimentally^13–16^ and in field applications^17^. While relatively low minimum effective vaccine virus titres (<10^8.0^ focus forming units (FFU)/mL) are needed for responsive species including foxes, raccoon dogs and mongooses (*Herpestes auropunctatus*)^18–23^, skunks and raccoons seem to be rather refractory to oral rabies vaccination, even when high virus titres were administered^13,16,24–28^. Also relatively high doses were needed to successfully immunise dogs (*Canis lupus familiaris*) by the oral route^14,29–33^.

Since the gastro-intestinal tract will rapidly inactivate and degrade the enveloped rabies virus, vaccine virus must be taken up in the oral cavity for the development of an immune response^34^. Despite the broad application of oral rabies vaccines over the last decades, it is still not fully understood if and where oral rabies vaccine viruses replicate in the oral cavity of the target species.

The Waldeyer’s ring is a ringed arrangement of lymphoid organs in the pharynx and consists of various tonsils^35^. In particular, the *t. palatina* is assumed to function as the main site for vaccine virus uptake and replication and therefore, appears to play a critical role in eliciting an effective immune response^36^. However, data are still sparse and the results obtained from the few experimental studies are contradictory. In foxes and dogs for example, the *t. palatina* was shown to be infected by attenuated rabies vaccine viruses^36–38^. However, these observations were contradicted by another study in which rabies vaccine virus could not be detected in the *t. palatina* of small Indian mongoose^39^. Also in the striped skunk, vaccine virus was less frequently detected after oral administration than in red foxes during a comparative study^36^. The latter findings suggested less efficient uptake or infection by vaccine virus in the *t. palatina* leading to insufficient immunity to rabies in this reservoir species^16,26–28,40–42^.

Against the background of the biological diversity among reservoir species for rabies involving representatives of the families of *Canidae*, *Procyonidae*, *Herpestidae*, *Mephitidae*, *Viverridae*, and *Mustelidae*^43^ and the lack of knowledge regarding species-specific vaccine uptake in the oral cavity, further studies are needed to investigate vaccine virus entry and replication in the *t. palatina* of those species^36^. Therefore, in this study our primary objective was to elucidate the detailed time course of vaccine virus infection in the *t. palatina* of the most important rabies reservoir species, e.g. red foxes, raccoon dogs, mongooses, raccoons, dogs and skunks, after oral application by conducting comparative experimental *in vivo* tracking studies. To this end, we used a highly attenuated and high titred GFP-labelled vaccine virus construct, followed by confocal laser-scan microscopy to visualize and assess differences between various important reservoir species. Prior to this full comparative study, we performed a pilot study to confirm that the results obtained with an attenuated vaccine virus strain^36^ were reliable using the genetically modified virus. Another objective was to answer the question whether tissues of the oropharyngeal tract other than the *t. palatina* are also involved in mediating immunity after oral vaccination by detecting the presence of viral RNA and viable virus using highly sensitive molecular diagnostic techniques. To this end, we analysed the anatomical and histological structure of the mucosa-associated lymphoid tissue (MALT) and Waldeyer’s ring to detect whether differences observed could have an impact on vaccine uptake efficiency.

## Results

### Restricted replication and limited spread of vaccine virus (SAD L16 GFP) infection in the *t. palatina* after oral inoculation of red foxes

**Figure 1:**
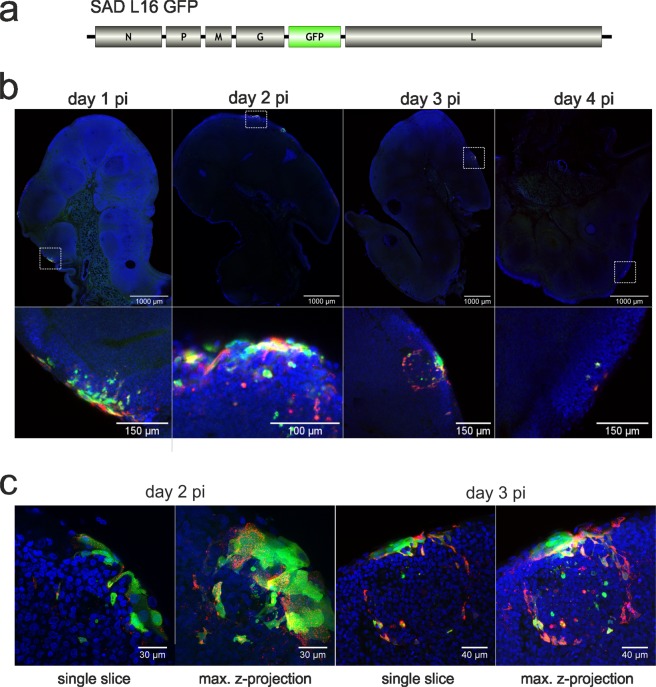
Spatio-temporal resolution of SAD L16 GFP infection in the *t. palatina* of foxes at day 1–4 post inoculation. (**a**) Genome organisation of the virus construct SAD L16 GFP. (**b**) Detection of virus infected cells in 150 µm vibratome slices by GFP auto-fluorescence (green) and immunostaining for RABV nucleoprotein N (red). Blue: Nuclei stained with Hoechst 33342. Top: Mosaic overview images generated from confocal tile scans performed with at low magnification (20x objective). Bottom: details from mosaic images shown. (**c**) Higher resolution images of individual infection foci at days 2 and 3 pi. Shown are single optical slices (left side) and maximum z-projections of confocal z-stacks.

Previous studies demonstrated that the *t. palatina* is a main target tissue for infection by orally administered RABV vaccines^36–38^. In the pilot study, we focused on the red fox as the species, which is very responsive to oral rabies vaccination. Here, we proved the suitability and functionality of the Green-Fluorescence-Protein (GFP) expressing model vaccine virus (SAD L16 GFP; Fig. 1a) for *in vivo* tracking to closely follow the kinetics and spread of vaccine virus in the oral cavity immediately after application.

In the pilot as well as in the full comparative study using a standardised approach, foxes inoculated orally with a GFP expressing vaccine virus were screened for the presence of vaccine virus at day 1, 2, 3, 4 and 10 post inoculation (pi). Confocal laser-scan microscope analysis was performed to detect both, GFP and RABV N protein, in vibratome slices of retropharyngeal tissues. Whereas GFP expression was not detectable in mucosa, tongue or lymph node tissues at any time point, GFP positive cells were present in all tonsils taken at day 1 to 4 pi (Fig. 1b). While at days 1 to 3 pi larger foci of infection were predominant, at day 4 pi only single GFP and N protein positive cells could be detected (Fig. 1b).

Already at day 1 pi, infection foci were detected in peripheral cell layers of the tonsils (Fig. 1b). In the following days pi, the size of infection foci in the epithelial cell layers did not increase and the virus did not infect deeper follicular and parafollicular areas of the lymphatic tissue, suggesting restricted replication and very limited spread of vaccine virus infection in of foxes. RABV N protein was associated with GFP positive cells (Fig. 1b) at day 1 pi, confirming that green fluorescence observed was proof of virus infection. When having a closer look at later time points (day 2, 3 pi), the ratio of GFP and N protein specific signals shifted towards increased detection of N protein (Fig. 1c), suggesting different kinetics of GFP and N protein accumulation and turnover in infected cells.

### Vaccine virus tropism and infection of the *t. palatina* differs among various target species

Since there is evidence that the efficacy of oral rabies vaccination varies between reservoir species, we compared the tropism of SAD GFP virus in the *t. palatina* after oral inoculation between six reservoir species. We classified species by whether or not they require a low dose (red fox, raccoon dog and small Indian mongoose) or a high dose (raccoons, dogs and striped skunks) for successful vaccination, based on previous literature. These species were inoculated using the same standardized approach described.

**Figure 2:**
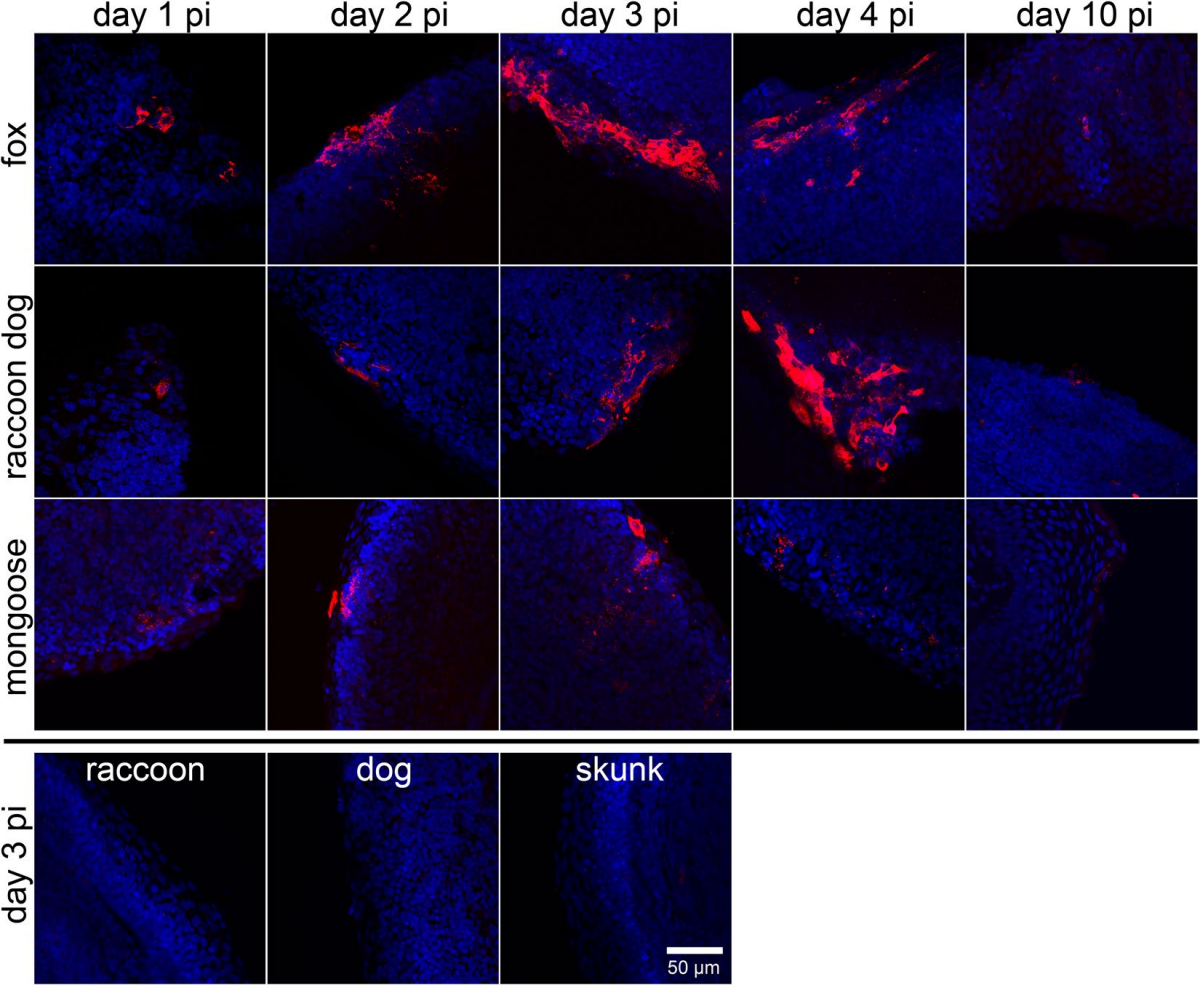
Spatio-temporal resolution of SAD L16 GFP infection in foxes, raccoon dogs, mongooses, raccoons, dogs and skunks by detection of the RABV nucleoprotein detection in the *t. palatina*. Comparative detection of virus infected cells in vibratome tonsil slices by nucleoprotein specific immunofluorescence (red) at time of necropsy (day 1–4, 10 pi). N protein detection failed for tonsil slices of raccoons, dogs and skunks at any time point (exemplary shown for day 3 pi). Blue: cell nuclei. Maximum projections of confocal z-stacks are shown.

Similar to the observation in foxes (Fig. 2, upper line), in other responsive species such as raccoon dogs and mongooses vaccine virus N protein specific fluorescence was detected in *t. palatina* until day 4 pi (Fig. 2, Table 1). No foci but only single vaccine virus infected cells were detectable at day 10 pi. In contrast, in the great majority of analysed tonsils of rather refractory species, i.e. raccoons, dogs and skunks, no vaccine virus specific signals were observed by laser-scan microscope imaging (Table 1), suggesting that infection of tonsil tissues in these species was strongly limited or did not even occur.

**Table 1:** Detection of RABV nucleoprotein in the *t. palatina* by immunofluorescence using confocal laser-scan microscopy post inoculation.

	day 1 pi		day 2 pi		day 3 pi		day 4 pi		day 10 pi	
individual animal	A	B	A	B	A	B	A	B	A	B
fox	−	++	+++	+	+++	++	+++	++	+	−
raccoon dog	+	+	−	++	−	+++	+++	+++	+	+
mongoose	−	+	−	++	−	++	−	++	−	+
raccoon	−	−	−	−	−	−	+	−	−	−
dog	+	+/−	−	+/−	−	−	−	−	−	+
skunk	−	−	−	−	−	−	−	−	−	−

At least three slices per tonsil and animal (A,B) were analysed. ++/+++: infection foci; +: single positive cells; +/−: signals questionable; −: no detection of infection foci or single infected cells.

**Figure 3:**
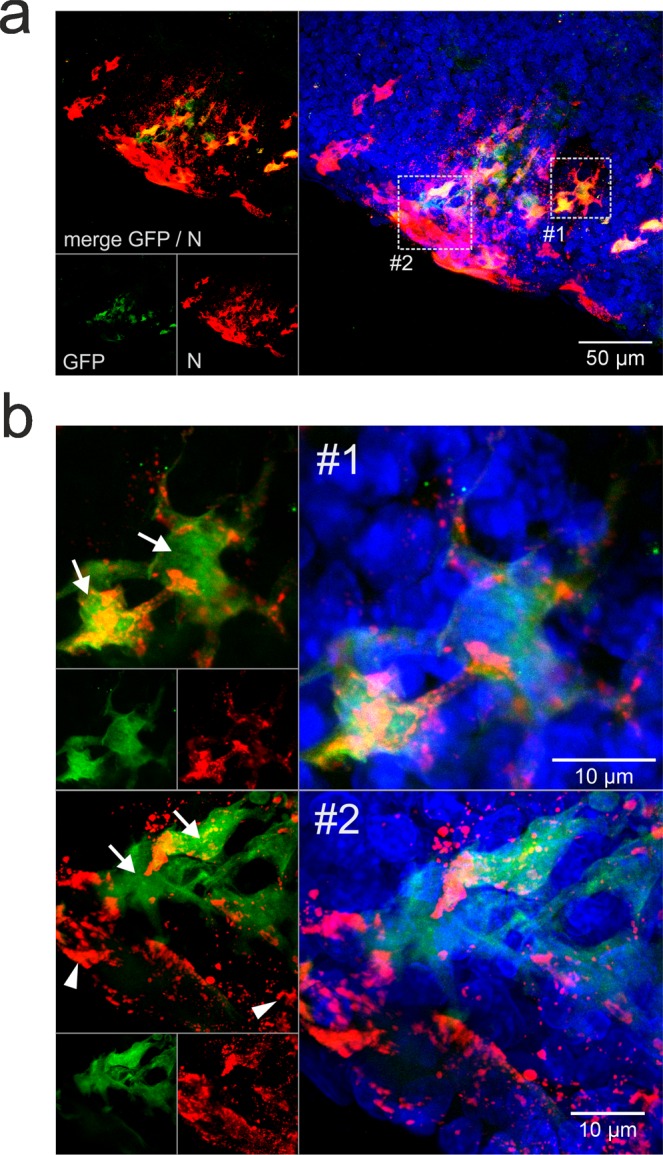
SAD GFP virus infection of fox *t. palatina* at 2 days post inoculation. (**a**) Focus of virus infected cells at day 2 pi. Maximum z-projection of a confocal z-stack. Green: GFP. Red: nucleoprotein (N). Blue: nuclei stained with Hoechst 33342. Dotted line boxes indicate in B shown details #1 and #2. (**b**) Details from A showing GFP positive cells with N signals (white arrows) and N signals without detectable GFP signals (arrowheads).

Similar to the pilot experiment depicted in Fig. 1, partial separation of GFP auto fluorescence and virus N protein specific immunofluorescence was observed, as shown by an infection focus in a fox *t. palatina* at day 2 pi (Fig. 3a). The details show GFP positive cells with moderate N detection (Fig. 3b, arrows) and cells with stronger N signals but without detectable GFP fluorescence (Fig. 3b, arrowheads).

### Analyzed carnivore species have a comparable anatomic configuration of Waldeyer’s ring, with minor variations in MALT

In a next step, we wanted to elucidate whether the differences observed in vaccine uptake efficiency among species can be explained by anatomical and histological differences in the morphological structure of the Waldeyer’s ring and MALT. The full comparative study started with the skunks, and a standard necropsy technique for preparation of tongue and adnexa of the Waldeyer’s ring was performed. However, this resulted in suboptimal representation of the anatomical features of Waldeyer’s ring. Therefore, we decided to variate the necropsy technique as described in materials and methods and showed in Supplementary Fig. S2 for optimal results.

All species had a comparable anatomic configuration of Waldeyer’s tonsillar ring. The current literature mainly focuses on the Waldeyer’s ring of dog species where a *tonsilla (t*.*) lingualis*, *t. palatina and t*. *pharyngea* can be differentiated^44^. This information is lacking for other carnivore species, therefore we comparatively investigated the presence or absence of MALT in all studied species (see Supplementary Table S1). Notably, in all studied carnivores, the *t. palatina* was the most prominent lymphoid structure (see Supplementary Fig. S2), followed by the *t*. *pharyngea* that was readily seen dorsocaudal of the opening of the eustachian tube as a patchy area with visible lymphoid follicles, except in the mongoose (see Supplementary Fig. S2). Histologically the entire structure is covered by a respiratory epithelium with an almost continuous basement membrane rarely infiltrated by lymphocytes and macrophages. A *t*. *veli palatina* or *t*. *lingualis* was not detectable in any of the studied carnivore species applying the criteria for MALT. Non-keratinised stratified squamous epithelium and a thin layer of respiratory epithelium on the nasopharyngeal side oro-pharyngeally covered the soft palate.

The *t. palatina* in each individual was covered by a non-keratinized squamous epithelium extensively infiltrated by lymphocytes and macrophages (lymphoepithelium). In the underlying submucosa variable prominent secondary lymphoid follicles with macrophages and interfollicular zones were seen (see Supplementary Fig. S3, left panel side). In all species, a regular structural morphology could be observed consisting of a variable thick multilayered cytokeratin positive non-keratinising squamous epithelium. Many CD20 positive B cells, fewer CD3 positive T cells and IBA1 positive macrophages, some with a dendritic cell morphology, infiltrated the lymhoepithelium (see Supplementary Fig. S3, right panel side). Frequently, interwoven nest or pockets of variable combinations of the aforementioned immune cells were observed within the squamous epithelium. RABV-nucleoprotein-positive cells were evident and predominantly confined to the non-keratinizing epithelium in 2 out of 2 foxes at 1 dpi, and in 1 out of 2 foxes at 2 and 3 dpi, respectively. Comparatively, RABV-antigen was detectable in epithelial cells in 1 out of 2 raccoon dogs at 2 dpi, and in 2 out of 2 raccoon dogs at 3 dpi. The underlying submucosal architecture showed no obvious species specific differences in the staining pattern of B cell-rich lymphoid follicles and T cell-dominated interfollicular zones.

### Relative viral load in the *t. palatina* is moderate to low depending on the target species

**Figure 4:**
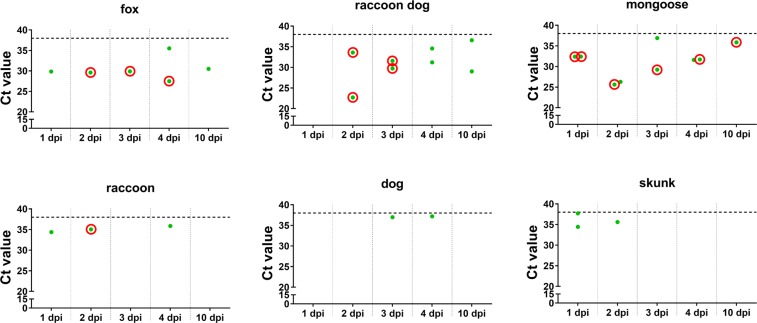
Detection of SAD L16 GFP in the *t. palatina* of different reservoir species by RT-qPCR. All animals received 10^8.0^ FFU/mL SAD L16 GFP by direct oral instillation. SAD L16 GFP positive (green dots): Ct values < 38, SAD L16 negative: Ct values ≥ 38 (dotted line, values not shown). *t. palatina*, which were tested positive for RABV RNA in RT-qPCR screenings, were tested for infectious virus by RTCIT. Samples for which infectious vaccine virus could be isolated are highlighted with a red circle.

In order to establish a correlation between the vaccine virus tropism and infection in the *t. palatina* as observed by confocal laser-scan microscopy in the different target species (Fig. 2, Table 1), we investigated the presence of viral RNA. Vaccine virus RNA could be detected in the *t. palatina* of more responsive species, i.e. foxes, raccoon dogs and mongooses, at almost all time points pi as opposed to raccoons, dogs and skunks (Fig. 4, Supplementary Table S2). With 31.04 the mean ct-value as a surrogate for the relative viral load was significantly lower (p < 0.0004) for the more responsive group of species as compared to the rather refractory species (mean ct-value 35.89), corroborating findings of the immunofluorescence analyses. Along with the findings from RT-qPCR screenings, infectious virus could only be isolated from the *t. palatina* of species more responsive to oral vaccination with the exception of one raccoon at day 2 pi (Fig. 4). For foxes, viable virus was detectable from 2 to 4 dpi and for raccoon dogs at days 2 and 3 pi. Detection of viable virus was highest in the *t. palatina* of mongooses as infectious virus could be isolated at all time points.

### Positivity rates for the detection of vaccine virus RNA in other tissues of the oropharyngeal tract also follow the same species-specific pattern

**Figure 5:**
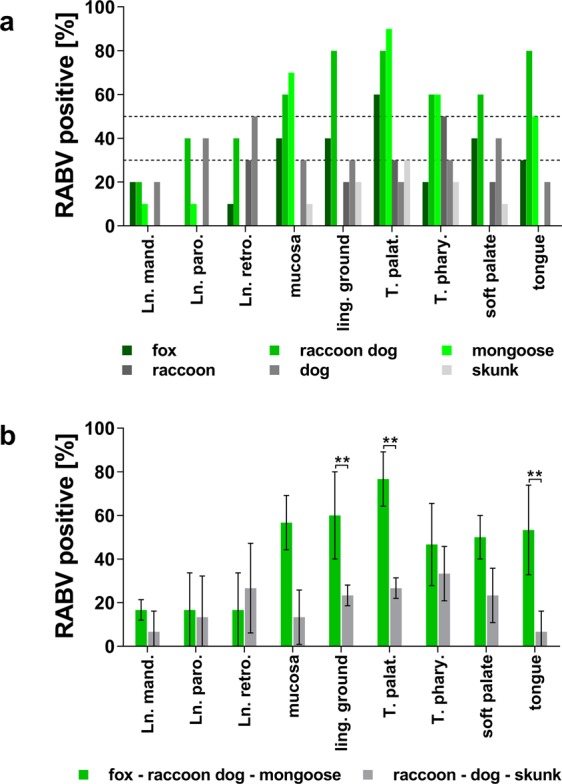
Positivity rates of tissue samples tested by RT-qPCR for the presence of viral RNA. (**a**) The columns show the percentage of positive samples per species and tissue. For each species, all time points were summarised (5 time points × 2 animals, n = 10). For better orientation, dotted horizontal lines were drawn at 50% and 30%. (**b**) Mean positivity rates for viral RNA as demonstrated by RT-qPCR. Species were grouped according to their assumed susceptibility to oral vaccination in responsive (fox-raccoon dog-mongoose = green) and refractory (raccoon-dog-skunk = gray) species. For each group, mean rates of RT-qPCR positive tissues (mean ± s.d.; n ≥ 2) are shown. Analyses of significance in the differences between two means were calculated by two-way ANOVA followed by Šidák’s multiple comparison test. Differences between two means with *p* < 0.01 were considered highly significant (**). Tissue abbreviation: Lnn. mand.- *lymphnodi mandibulares*, Ln. paro. – *lymphnodus parotideus*, Ln. retro. – *lymphnodus retropharyngealis*, ling. ground – lingual ground, T. palat. – *tonsilla pallatina* (elsewhere referred to as *t. palatina*), T. phary. – *t*. *pharyngea*,.

To investigate whether tissues other than the *t. palatina* are also involved in vaccine virus uptake, nine tissues of the oropharyngeal tract were screened by RT-qPCR. Viral RNA could be detected in all tissues, albeit with differences in positivity rates. High positivity rates were observed in the *t. palatina*, followed by *t*. *pharyngea*, mucosa, tongue, and lingual ground tissue (Fig. 5a). The *t. palatina* of foxes, raccoon dogs and mongooses revealed a higher positivity rate and lower Ct-values (Fig. 5a, Supplementary Tables S4–S6) as opposed to raccoons, dogs and skunks (Fig. 5a, Supplementary Tables S7–S9). These data may indicate that both the frequency of vaccine virus infection and the relative viral load was increased in the *t. palatina* of foxes, raccoon dogs and mongooses compared to the other species.

When species were combined according to their assumed responsiveness to oral rabies vaccination, irrespective of the time point after inoculation, the overall positivity rate across all tissues in responsive species (54%), i.e. foxes, raccoon dogs and mongooses, was higher as opposed to rather refractory species (32%), i.e. raccoons, dogs and skunks, although not statistically significant. In contrast, differences were observed for *t. palatina*, mucosa and tongue tissues showing significantly (p < 0.01) higher positivity rates in responsive species as compared to rather refractory species (Fig. 5b, Supplementary Table S3). Even though viral RNA was also detected in samples from lymph nodes, positivity rates did not exceed 30% in most species (Fig. 5a).

### Viable vaccine virus was only detectable up to four hours post inoculation, while RNA was present up to four days post inocluation in oral swabs

In order to investigate the longevity of viral RNA and viable virus in the oropharyngeal cavity and to see whether detection of the oral rabies vaccine virus in the oropharynx and infection of particular target cells or tissues is a determinant for vaccine uptake, oral swabs were collected 2, 4 and 24 hrs pi and at the day of euthanasia. Virus detection by RT-qPCR was generally highest in oral swabs taken immediately after direct vaccine virus administration (Table 2). While except for skunks, almost all samples taken 2 hrs pi were positive for viral RNA, after 4 hrs virus detection decreased. At this time point, only oral swab samples of foxes, raccoons and dogs were still virus RNA positive, whereas the positivity rate in raccoon dogs, mongooses and skunks ranged between 30% and 70%. Sporadically, viral RNA could be detected in individuals of several species up to day 4 pi (Table 2). On the opposite, viable virus could only be found up to 4 hrs pi. Notably, virus could only be isolated from three oral swabs from skunks and no viable virus was detected in any of the oral swabs from dogs, whereas all other species had at least 12 oral swabs each during the first 4 hours with positive results (Table 2). Interestingly, almost all saliva specimen of raccoons within the first 4 hrs were positive for viable virus. This viable virus cannot be a result of active shedding of progeny vaccine virus but is merely vaccine virus administered and not yet cleared from the oral cavity.

**Table 2:** Detection of vaccine virus (ct values) in oral swabs by RT-qPCR and RTCIT.

Time p.i.	2 h	4 h	1 d	2 d	3 d	4 d	10 d	Time p.i.	2 h	4 h	1 d	2 d	3 d	4 d	10 d
**RT-qPCR (ct-values) and virus isolation in RTCIT**															
Fox_01	**22.2**	29.2	—	nd	nd	nd	—	RC_01	**22.2**	**25.4**	33.8	nd	nd	nd	—
Fox_02	**22.2**	33.2	—	nd	nd	nd	—	RC_02	**26.6**	**34.2**	—	nd	nd	nd	—
Fox_03	**19.6**	29.9	—	nd	nd	28.0		RC_03	**30.3**	31.0	—	nd	nd	30.7	
Fox_04	**22.8**	**24.2**	—	nd	nd	34.8		RC_04	27.5	31.4	32.0	nd	nd	—	
Fox_05	**21.1**	29.6	—	nd	—			RC_05	**26.0**	**29.3**	—	nd	—		
Fox_06	**20.2**	30.9	—	nd	32.6			RC_06	**31.6**	**31.8**	—	nd	—		
Fox_07	**21.4**	33.4	—	—				RC_07	**33.8**	**29.4**	—	—			
Fox_08	**27.4**	33.5	—	—				RC_08	**28.5**	**30.5**	—	—			
Fox_09	**27.8**	37.9	36.1					RC_09	**27.2**	**27.7**	—				
Fox_10	**24.3**	32.6	35.2					RC_10	31.1	29.7	34.5				
RD_01	**28.6**	30.4	—	nd	nd	nd	—	Dog_01	32.0	31.7	—	nd	nd	nd	—
RD_02	30.9	**32.6**	—	nd	nd	nd	—	Dog_02	32.4	33.3	35.4	nd	nd	nd	—
RD_03	**28.5**	—	—	nd	nd	—		Dog_03	30.3	31.5	32.8	nd	nd	—	
RD_04	26.0	—	—	nd	nd	—		Dog_04	33.9	36.2	—	nd	nd	37.4	
RD_05	**27.1**	**36.5**	—	nd	—			Dog_05	32.4	35.3	—	nd	—		
RD_06	**34.9**	—	—	nd	—			Dog_06	33.8	33.0	—	nd	—		
RD_07	**28.6**	—	—	—				Dog_07	32.0	30.4	37.0	—			
RD_08	**26.0**	**30.1**	—	—				Dog_08	31.3	34.4	—	—			
RD_09	28.7	—	—					Dog_09	30.0	34.4	37.4				
RD_10	**30.9**	32.2	—					Dog_10	27.0	30.2	—				
MG_01	**29.5**	—	—	nd	nd	nd	—	SK_01	36.1	35.2	—	nd	nd	nd	—
MG_02	**30.0**	—	—	nd	nd	nd	—	SK_02	30.2	32.8	36.0	nd	nd	nd	—
MG_03	—	—	—	nd	nd	—		SK_03	—	35.0	—	nd	nd	—	
MG_04	**29.3**	—	—	nd	nd	—		SK_04	—	36.5	—	nd	nd	—	
MG_05	**28.2**	—	32.8	nd	—			SK_05	**30.7**	34.7	—	nd	—		
MG_06	**21.9**	**33.5**	—	nd	35.5			SK_06	**30.0**	37.4	37.0	nd	—		
MG_07	**20.0**	**30.5**	—	—				SK_07	36.7	—	36.0	—			
MG_08	**27.8**	—	—	—				SK_08	—	—	35.7	—			
MG_09	**30.6**	**35.4**	28.8					SK_09	**32.4**	—	—				
MG_10	**28.1**	—	36.9					SK_10	—	31.3	—				

All animals received 10^8.0^ FFU/mL by direct oral instillation. −: negative; nd: not determined; blank space: animals already euthanised. Oral swabs, which were positive for RABV RNA in RT-qPCR screenings, were tested for infectious virus by RTCIT. Samples for that infectious vaccine virus could be isolated are marked in bold. Animal abbreviation: RD – raccoon dog, MG – mongoose, RC – raccoon, SK – skunk.

### All animals regardless of species produced RABV specific antibodies by day 10 post inoculation

To see whether oral application of the GFP labelled vaccine virus strain SAD B19 elicited a measurable immune response, the presence of rabies specific antibodies was tested by two different diagnostic assays.

All animals were naïve at the time point of vaccine virus application as demonstrated by the absence of rabies specific antibodies as measured both by RFFIT and ELISA. Notably, at day 10 pi all animals had seroconverted as indicated by ELISA (Table 3), and with exception of one skunk, all animals exhibited virus neutralising antibody (VNA) titres above the threshold of 0.5 IU/ml. When reservoir species were grouped according to the presumed responsiveness to ORV a significant difference between more responsive, i.e. foxes, raccoon dogs and mongooses, as opposed to the remaining less responsive species could only be observed for VNA titres (Table 3).

**Table 3:** RABV specific VNA and binding antibodies in serum samples at day 0 and 10 post inoculation.

	**days pi**							
	**0**				**10**			
	VNA (RFFIT) [IU/mL]		RABV Ab (ELISA) [% inhibition]		VNA (RFFIT) [IU/mL]		RABV Ab (ELISA) [% inhibition]	
fox 1	<0.5	**−**	16.16	**−**	7.65	**+**	76.94	**+**
fox 2	0.33	**−**	38.58	**−**	70.68	**+**	90.94	**+**
raccoon dog 1	0.08	**−**	12.24	**−**	9.66	**+**	59.21	**+**
raccoon dog 2	0.25	**−**	18.42	**−**	0.57	**+**	46.40	**+**
mongoose 1	0.19	**−**	15.28	**−**	45.03	**+**	61.25	**+**
mongoose 2	0.15	**−**	12.61	**−**	23.56	**+**	68.86	**+**
raccoon 1	0.03	**−**	23.54	**−**	2.39	**+**	68.99	**+**
raccoon 2	0.06	**−**	28.77	**−**	1.16	**+**	60.12	**+**
dog 1	0.02	**−**	12.19	**−**	0.55	**+**	42.78	**+**
dog 2	0.25	**−**	13.65	**−**	1.19	**+**	41.08	**+**
skunk 1	0.06	**−**	21.97	**−**	1.38	**+**	75.30	**+**
skunk 2	0.05	**−**	9.96	**−**	0.04	**−**	54.43	**+**

VNA (RFFIT) <0.5 IU/mL/RABV Ab (ELISA) <40% inhibition = no seroconversion (−), VNA (RFFIT) ≥ 0.5 IU/mL/RABV Ab (ELISA) ≥40% inhibition = seroconversion (+).

## Discussion

Oral rabies vaccination of wildlife is a challenge as there are a variety of reservoir species that need to be targeted^45^. By coincidence, the red fox, the initial species for the development of the concept of oral rabies vaccination, was also the species that was highly susceptible to oral rabies vaccination; a relative low minimum effective dose was needed to elicit a protective immune response^19–21,46^. It was only by experience from experimental and field data that other reservoir species required much higher doses to be successfully immunised by the oral route. Principally, due to the instability of the rabies virus, the gastro-intestinal tract will cause rapid antigen degradation, and hence the rabies virus vaccine must be taken up in the oral cavity for the development of an immune response^34^. However, from some of these early studies in foxes, it became clear that orally administered inactivated rabies virus vaccines did not induce protective immunity^47,48^, indicating that vaccine virus replication within the host was essential. Hence, presently all available oral rabies vaccines are live replication-competent virus constructs. Based on our previous findings^36^, this study aimed at elucidating the species-specific uptake, distribution and kinetics of oral rabies vaccines in the oral cavity of the most important terrestrial rabies reservoir species in order to identify barriers for immunisation with a focus on species known to be rather refractory to oral vaccination. To this end, in this first comparative and comprehensive *in vivo* tracking study with a standardised approach, we used a genetically modified vaccine virus construct, followed by up-to date techniques in imaging and viral RNA detection. Earlier studies could identify infected cells^36,37^. In this study, using the SAD L16 GFP construct we were also able to identify cells in which active virus gene expression took place.

Our data confirm that orally administered rabies vaccine virus multiplies at a low level within the oral cavity of foxes, particularly, but not exclusively in the *t. palatina*, as shown before^37^. When analysing the time-course of infection in the *t. palatina*, immunofluorescence analyses of viral nucleoprotein and GFP fluorescence revealed foci of RABV infected cells in the peripheral cell layers of fox tonsils at days 1 to 4 pi (Figs. 1b and 2). These data confirmed previous snapshots of SPBN GASGAS vaccine virus infection of red fox peripheral tonsil layers^36^ and demonstrate the utility of GFP-labelled vaccine virus strains for *in-vivo* tracking. Limitation of both vertical and lateral spread of virus infection, strongly indicate a spatio-temporal restriction of vaccine virus tropism and replication in tonsils of foxes.

To follow vaccine virus uptake and subsequent tropism and time course of infection in the *t. palatina,* next to GFP auto fluorescence, we additionally focused on N protein staining. The observed phenomena of accumulation of N protein aggregates and loss of GFP fluorescence in fox tonsils from day 2 onwards (Figs. 1c and 3b) clearly suggest different kinetics of GFP and N protein accumulation and turnover in vaccine virus infected cells. The N protein as part of intracellular ribonucleoproteins (RNPs) accumulates in large inclusion bodies and is likely to be less prone to degradation, while soluble GFP may be less stable in infected cells and may be rapidly removed by host immune system.

Strikingly, comparative immunofluorescence analyses revealed substantially more vaccine virus and virus infected cells in species that are more responsive to oral vaccination, thus corroborating general assumptions and field observations on differences among reservoir species in vaccine uptake efficiencies^8,17,49–52^ and responsiveness to oral rabies vaccination^18–23^. Viral N-protein staining confirmed locally restricted areas of RABV infected cells in the peripheral layer of the *t. palatina* of foxes, raccoon dogs and mongooses with similar limited vertical and lateral spread of virus infection, but failed to identify even single infected cells in the other species (Fig. 2, Table 1). These observations were corroborated by the differences in the detection of infectious virus and relative viral load in the *t. palatina* of the respective target species (Figs. 4 and 5). The reasons for these obvious differences remain elusive. As discussed before^36^, the morphology of the lymphoreticular tissue of the pharynx cannot explain the observed differences in vaccine uptake among the species studied here. All species had a comparable anatomic configuration of Waldeyer’s tonsillar ring with a dominating *t. palatina*. Also histologically, there was no difference in the cellular structure of the tonsils, which were represented by a peripheral non-keratinised stratified squamous epithelial cell layer followed by germinal centres consisting of lymphocytes (see Supplementary Fig. S3).

In our study we were required to use a very high vaccine dose (10^8.0^ FFU/mL) to increase the likelihood to observe any differences in vaccine uptake in MALT of the oral cavity of the various species at all. This could be the reason why all animals regardless of species exhibited RABV specific antibodies by day 10 pi (Table 3) as measured by the standard RFFIT^53^ and the more sensitive ELISA^53,54^, irrespective of the identification of infected cells in the *t. palatina*. Therefore, other tissues in the oropharyngeal cavity must have been involved in virus uptake and subsequent interaction with the immune system. Screening for viral RNA demonstrated its presence in all investigated tissues, albeit with significant differences in the positivity rates (Fig. 5). The positivity rate for tonsils was higher for species known to be more responsive to oral vaccination as was the relative viral load (Fig. 5) corroborating findings of the immunofluorescence analyses (Figs. 1b and 2). Besides the *t. palatina*, significantly increased virus detection by RT-qPCR were also seen in mucosa and tongue samples of more responsive species (Fig. 5b), suggesting that infection of other oropharyngeal tissues also contributes to vaccine efficacy. However, based on our data there seems to be no preferential site for vaccine virus uptake and replication in low-responsive species. Our quantitative serological results suggest a correlation with responsiveness to oral vaccination in various species for RFFIT, whereby mean VNA titres of responsive species were significantly higher (p < 0.05) as opposed to low responsive species (Table 3). Even taking this limited number of animals into account this corroborates previous findings^53^.

The short time window (day 10 pi for virus RNA detection, day 4 pi for antigen detection) during which vaccine virus (SAD L16 GFP) could be detected in tonsils is noteworthy. When dissemination of a conventional C-strain vaccine or a modified live marker vaccine (CP7_E2alf) for classical swine fever in tissues was investigated, vaccine virus genomes were consistently detected in the tonsils lymphoid up to day 7 pi^55^, day 42 pi^56^ and day 77^57^ pi by RT-qPCR. The results indicate that, as with other oral vaccines, vaccine virus detection is usually transient even in lymphatic organs. However, in contrast to the other vaccines, duration time of the rabies vaccine in the tonsils is much shorter and the relative viral load in the *t. palatina* remains rather low (Figs. 1, 2 and 4)^56^.

In previous studies, detection of vaccine virus and RNA in oral swabs post vaccination was shown to be residual input virus and not virus shedding as such^36^. Notably, a clear distinction between responsive and refractory species as seen for vaccine virus tropism and infection in the *t. palatina* and other tissues in the oropharyngeal cavity was only partially visible when assessing the detection of SAD L16 GFP in oral swabs, partly contrasting earlier findings with the genetically engineered vaccine virus construct SPBN GASGAS^36^. In our study, vaccine virus could only be re-isolated from oral swabs within 4 hours except for dogs where only RNA was present (Table 2). The detection of vaccine RNA in this time window points to a degradation of input virus by the environment in the oral cavity. Occasional detection of vaccine virus RNA in oral tissues beyond day 1 pi (see Supplementary Tables S4–S9) may be due to release of non-infectious virus particles from infected cells or the detection of dislodged cells containing vaccine virus RNA.

Taken together, the data corroborate the hypothesis of rapid clearance of attenuated rabies virus vaccines in the oral cavity as described before regardless of the target species^37,58^. The mechanism behind this could be related to the activation of NFκB related genes, which might lead to a rapid clearance at the primary sites of infection as shown for SPBN GASGAS^59^. Still quite unknown is whether RABV infection of related immune cells, which could trigger a strong antigen specific response, influences the development of a protective immunity. *In vitro* and *in vivo* studies revealed that rabies virus could directly infect immune cells. RABV infection of human and mouse T lymphocytes induced apoptosis, which subsequently lead to an enhanced immune response by activating macrophages, cytokine cascades and increased antigen presentation^60^. Also, RABV was shown to infect and activate primary B cells, which subsequently directly primed and activated CD4^+^ T cells *in vitro*^61^. To investigate whether B and T cell infection occur *in vivo*, further functional characterisation of target cells, such as lymphocytes in the epithelial cell layers of infected tonsils, is required.

## Conclusions

The comparison of the *in vivo* tropism and time course of infection of an attenuated oral rabies vaccine virus in the oropharyngeal tract of the most important rabies reservoir species^43^ after direct oral instillation clearly revealed species-specific differences. Although the detailed mechanisms of vaccine virus uptake and processing as well as the involvement of potentially infected immune cells in the *t. palatina* need further clarification, the results strengthen the hypothesis that certain reservoir species appear more refractory to oral vaccination than others. Whether field effectiveness of ORV for example in skunks and raccoons appears to be limited by poor bait uptake or inadequate ingestion of vaccine rather than from poor vaccine efficacy^62^ remains to be proven.

Next to the *t. palatina* as a main site of vaccine virus uptake, other tissues in the oropharyngeal cavity may play a greater role in virus uptake and subsequent interaction with the immune system than assumed before. Understanding the mechanisms of vaccine virus uptake and replication is crucial for vaccine development and optimisation. Next to improved or new vaccines that lead to enhanced field performance^17^, further research should investigate how vaccine uptake efficacy in raccoons, skunks and other rather refractory reservoir species can be improved, for example by increasing vaccine titre, vaccination intervals or by adding muco-adhesive and/or permeation enhancing substances^63–66^ to allow for future elimination strategies.

## Material and Methods

### Virus

The laboratory strain SAD L16 is a recombinant full-length clone of the attenuated oral vaccine strain SAD B19^67,68^. A GFP expressing variant was generated by a standard rescue protocol^69^ after insertion of an additional transcription unit between virus genes G and L at SAD L16 genome position 5338. The inserted sequence comprised a duplicated N/P gene border sequences (SAD L16 nt positions 1413–1500) followed by an ORF coding for an EGFP protein with N- and C-terminal Strep- and His-tags, respectively.

### Ethics statement

All animals were kept in accordance with the prevailing guidelines and general care was provided as required. While the pilot fox study (42502-3-725) and the study in dogs (42502-3-762) was evaluated and approved by the Ethics Committee of the Federal State of Saxony Anhalt, Landesverwaltungsamt Sachsen-Anhalt, 06003 Halle, Germany, all other studies were evaluated and approved by the Ethics Committee of the Federal State of Mecklenburg-Western Pomerania, Landesamt für Landwirtschaft, Lebensmittelsicherheit und Fischerei Mecklenburg-Vorpommern, 18003 Rostock, Germany (7221.3-1-058/15).

### Animals

10 animals per species (foxes, raccoon dogs, mongooses, raccoons, dogs and striped skunks) were used for oral inoculation and dissemination studies.

Adult foxes, raccoon dogs, raccoons, skunks and dogs (breed: beagles HsdRcc: DOBE, 4–5 kg) were purchased from commercial breeders, whereas mongoose were caught using baited box traps on the rabies-free island Korčula, Croatia, and transported to Germany. Except for dogs and foxes of the pilot study, all animals were kept in individual stainless steel cages at 20 °C room temperature, 60–80% humidity and a 12 hr/12 hr (35% dimming during night modus) lighting control within a fan forced draught ventilation equipped BSL3** animal facility at the Friedrich-Loeffler-Institute (FLI), Greifswald – Insel Riems, Germany. Dogs and foxes of the pilot study were kept in single cages at a room temperature of 20–25 °C, 20–70% humidity and a 12 hr/12 hr light-dark control within the animal facility at the Ceva Innovation Center GmbH (Dessau-Roßlau, Germany). Animals were fed daily with commercially produced feed for farmed-kept foxes and raccoon dogs (Schirmer und Partner GmbH Co KG, Döhlen, Germany; Michael Hassel GmbH, Langenargen, Germany). The diet was supplemented with vitamins, minerals and items like 1-day old chicken. Water was offered ad libitum. The general health status of all animals, feed uptake and defecation was observed and recorded daily.

Blood samples were taken prior to immunisation and at day of necropsy (see Supplementary Fig. S1). For animals at the FLI (foxes of the comparative study, raccoon dogs, mongooses, raccoons and skunks), collection of blood samples and administration of virus were conducted under anaesthesia using Zoletil® (combination of Tiletamin and Zolazepam, Virbac, France). Blood was taken from the large superficial veins of the extremities (e.g. *Vena cephalica antebrachii*, *Vena saphena*). For euthanasia, animals were first anaesthetised with Zoletil® followed by cardiac bleeding and subsequent administration of T61® (Intervet, Germany). Foxes of the pilot study were anaesthetised with a combination of Xylazine and Ketamine whereas for dogs no anaesthesia for blood sampling and virus administration was needed. For euthanasia, foxes and dogs were first anaesthetised using a combination of Xylazine and Ketamine (foxes) and Medetomidine, Acepromazine and Butorphanol (dogs) followed by cardiac bleeding and subsequent administration of T61®.

### Oral inoculation and dissemination studies

To investigate species-specific differences in vaccine virus tropism, all animals received 1.0 mL SAD L16 GFP (10^8^ FFU/mL) by direct oral application (d.o.A.). This rather high dose was selected because it increased the likelihood of successful immunization in all species based on experience with SAD B19 or SAD B19-derived oral rabies virus vaccines^19,20,23,28,32^.

To prove the suitability and functionality of the Green-Fluorescence-Protein (GFP) expressing model vaccine virus and to follow the time course of vaccine virus infection in the *t. palatina* after oral application in a highly responsive species, in a pilot study (Fig. 1), two foxes were sacrificed 2, 3, 4 and 10 days and one animal 1 day post inoculation (pi). At necropsy, samples of the *t. palatina*, lymph node tissues, mucosa, and tongue were collected for immunofluorescence analysis.

In the subsequent comparative study (Fig. 2) aimed at elucidating differences in vaccine uptake efficiency, two animals of each species were sacrificed 1, 2, 3, 4 and 10 days pi, respectively (see Supplementary Fig. S1). At necropsy, the skulls were carefully dissected in two halves with a diamond band saw to expose Waldeyer’s ring and tongue. Subsequently, the following tissues were carefully prepared and samples of the lymph nodes (*lymphonodii (lnn*.*) mandibulares*, *lnn*. *parotidei*, *lnn*. *retropharyngei*), mucosa, tongue and parts of the Waldeyer´s ring (*lingual ground*, *t. palatina*, *t*. *pharyngea*, *soft palate*) were collected and screened for vaccine virus construct by RT-qPCR. One half of each skull with the Waldeyer’s ring attached was immediately fixed in 4% neutral-buffered formaldehyde for at least 14 days. Similar tissue samples as aforementioned were processed, embedded in paraffin wax and 2–4 µm sections were stained with hematoxylin and eosin (HE). Specimens were histologically assessed for the presence of lesions, and the occurrence of mucosa-associated lymphoid tissue (MALT) following standard criteria: variable interfollicular T-cell zones, multiple B-cell follicles, lack of afferent lymphatics, direct exogenous antigen sampling by mucosal surfaces with microfold/membrane (M) cells^35^ using an Axio Imager M2 microscope (Carl Zeiss Microscopy). For immunofluorescence analysis, tissues were fixed in 4% buffered formaldehyde (pH 7.4). Saliva swabs were taken prior to (0 h) and 2 h, 4 h and 24 h after oral RABV administration as well as during necropsy (see Supplementary Fig. S1). Saliva samples were collected by wiping off the oral cavity for at least 1 minute. The cotton tips were stored at −80 °C until subsequent analysis of virus construct by RT-qPCR and rabies tissue culture infection test (RTCIT).

### Diagnostic assays

For detection of RABV RNA in oropharyngeal tissue samples and saliva swabs, RNA was extracted fully automated using the MagAttract Viral RNA M18 Kit in combination with the BioSprint 96 Workstation (Quiagen, Hilden) according to manufacturer’s instructions. Subsequently, reverse transcription quantitative PCR (RT-qPCR) was done as previously described^70^. Positive tested saliva and *t. palatina* samples were further analysed for presence of infectious rabies virus by RTCIT^71,72^ using the cell line BHK-21 [BSR/5] (Collection of Cell Lines in Veterinary Medicine (CCLV), Friedrich-Loeffler-Institut, No. 0194). To confirm a negative result, three sequential cell culture passages were carried out.

For the detection of virus neutralising antibodies (VNA), collected blood samples were analysed by rapid fluorescent focus inhibition test (RFFIT) as described elsewhere^53^. Binding antibodies were detected by rabies virus specific inhibition ELISA according to manufacturer specifications (BioPro Rabies ELISA Ab kit, O.K. Servis BioPro, Prague, Czech Republic)^54^.

### Immunofluorescence analysis

For subsequent immunofluorescence analysis, vibratome sections of fixed tonsils with a thickness of 150 µm were prepared. Because of the small size, cryostat sections of mongoose *t. palatina* with a thickness of 20 µm were prepared and mounted on a slide. Tonsil sections were incubated overnight with a specific primary antibody against RABV nucleoprotein (polyclonal rabbit-α-RABV N 161-5^73^, diluted 1:3000 in 0.1% Triton/PBS), followed by an incubation with the fluorophore-coupled secondary antibody (Alexa Fluor^®^ 568 goat-α-rabbit, 0.7 µg/mL in 0.1% Triton/PBS, ThermoFisher Scientific) for 4 h. Nuclei were visualised with Hoechst 33342 (1 µg/mL in PBS, ThermoFisher Scientific). Stained *t. palatina* slices were documented using the confocal laser-scan microscope Leica DMI 6000 TCS SP5 with a 63-fold oil immersion objective (Leica Microsystems).

### Immunohistochemistry

To characterize the cellular morphology of *t. palatina* in each species, immunohistochemistry was applied using the avidin-biotin-peroxidase-complex (ABC) method utilizing the Vectastain® Elite ABC standard kit (Vector Laboratories) with citric buffer (10 mM, pH 6,0) pre-treatment, to label the following tonsillar epitopes: cytokeratins 1–8, 10, 13–17, 19 (clone AE1/AE3, mouse anti-human, monoclonal, diluted 1:500, Dako, Deutschland GmbH, Hamburg, Germany), macrophages and dendritic cells (anti IBA1, rabbit anti-human, polyclonal, diluted 1:200, FUJIFILM Wako Chemicals, Germany), B cells (CD20, rabbit anti-human, polyclonal, diluted 1:200, Thermo Fisher, Germany), T cells (CD3, rabbit anti-human, polyclonal, diluted 1:200, Dako, Germany,) and RABV-nucleoprotein (polyclonal, rabbit-α-N161-5; diluted 1: 2000^73^) by incubating overnight. Antigen visualization was performed with 3-amino-9-ethyl-carbazol as chromogen and hematoxylin as counterstain. As negative controls, consecutive sections were incubated with rabbit serum or tris-buffered saline instead of the primary antibodies.

### Statistical analysis

Statistical significance in the differences between two means (RT-qPCR positivity) between various lymphoid tissues in responsive versus low-responsive species was assessed by two-way ANOVA followed by Šidák´s multiple comparison test. Other data including the overall RT-qPCR positivity, the ct-values in *t. palatina*, and serological results were tested for significance using an unpaired T-test. Analyses were carried out using Graphpad Prism 7 (GraphPad Software Inc., San Diego, CA, USA), with P values < 0.05 considered statistically significant and P values < 0.01 considered highly significant, respectively.

## Supplementary information

Supplementary Information.

## Data Availability

All data generated or analysed during this study are included in this published article (and its Supplementary Information Files).
