# Supplementary material for: Responsiveness of various reservoir species to oral rabies vaccination correlates with differences in vaccine uptake of mucosa associated lymphoid tissues
Source: Sci Rep. 2020 Feb 19;10:2919. doi: 10.1038/s41598-020-59719-4 (PMC7031338; doi:10.1038/s41598-020-59719-4)
Supplement: Supplementary file 1 — Supplementary Information. [file 41598_2020_59719_MOESM1_ESM.docx]

**Responsiveness of various reservoir species to oral rabies vaccination correlates with differences in vaccine uptake of mucosa associated lymphoid tissues**

Verena te Kamp, Conrad M. Freuling, Ad Vos, Peter Schuster, Christian Kaiser, Steffen Ortmann, Antje Kretzschmar, Sabine Nemitz, Elisa Eggerbauer, Reiner Ulrich, Jan Schinköthe, Tobias Nolden, Thomas Müller, Stefan Finke

**Supplementary Information**

**Supplementary Figures**

**
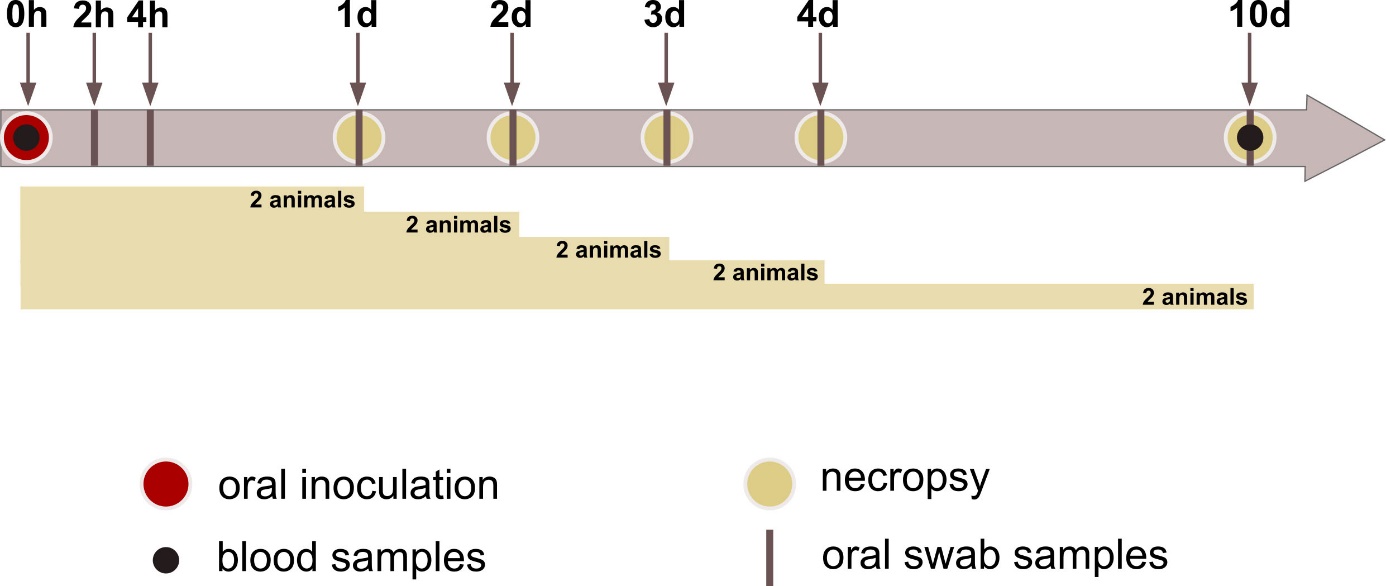
**

**Supplementary Figure S1: Experimental design and scheme of sampling.** A total of ten animals per species were orally inoculated with 10^8^ FFU/mL SAD L16 GFP. Two animals per species were euthanized 1, 2, 3, 4 and 10 days pi, respectively (necropsy). After 2 and 4 hours pi and at day of necropsy oral swab samples were taken. For determination of RABV specific Ab, blood samples were collected at the day of inoculation (0h) and 10 days pi.


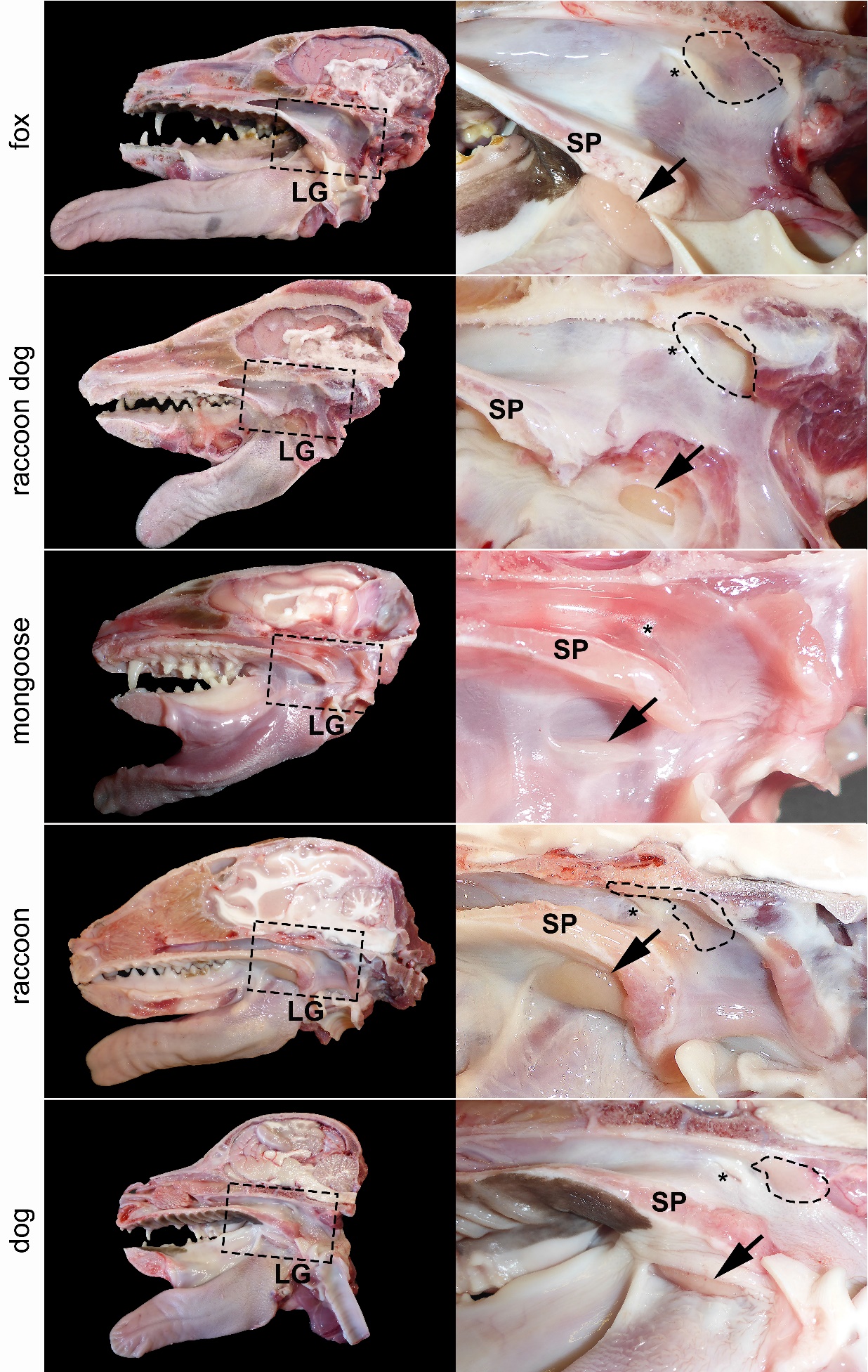


**Supplementary Figure S2: Gross anatomical features of the Waldeyer‘s ring of different wild and domestic carnivore species.** Left panel side: Macrophotographs show carefully dissected skull halves with the Waldeyer‘s ring, mandibula and tongue. Right panel side: Focused areas of the dashed rectangles in the left panel side representing *tonsilla palatina* (arrows), opening to the eustachian tube (asterisks) and *tonsilla pharyngea* (dashed lines). LG, lingual ground; SP, soft palate.


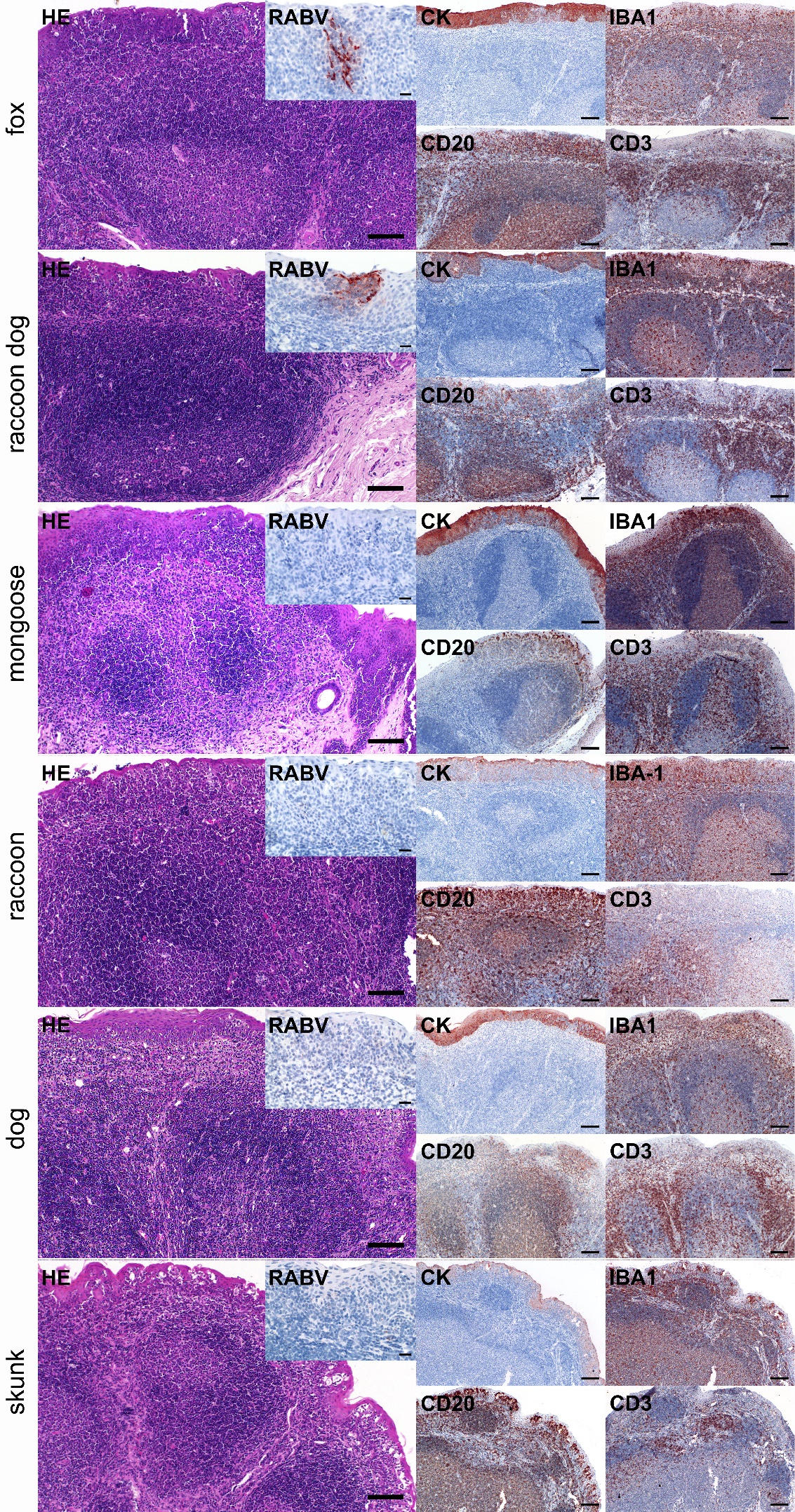


**Supplementary Figure S3: Immunohistochemical analysis of cellular epitopes and RABV N-protein in *t. palatina* of different wild and domestic carnivore species.** Left panel side: Each section shows hematoxylin and eosin (HE) stained *t. palatina* in variable degrees of activation concerning the lymphoepithelial and submucosal lymphoid tissue. Insets: For foxes and raccoon dogs, RABV N-protein were detectable, while detection of RABV antigen failed in other species. Right panel side: Photomicrographs indicate the spatial localization of cytokeratine (CK), macrophage/microglia specific ionized calcium-binding adapter molecule 1 (IBA1), B-lymphocyte antigen (CD20) and the T cell co-receptor protein complex (CD3). Bars: HE-stained and CK, IBA1, CD20, CD3 labeled sections: 100 µm; RABV N-protein stained sections: 20 µm.

**Supplementary Tables**

**Supplementary Table S1. Semiquantitative assessment of presence or absence of mucosa-associated lymphoid tissue (MALT) in the Waldeyer’s ring in different carnivore species.**

| **species** | **soft palate** | **lingual ground** | ***t. palatina*** | ***t. pharyngea*** | ***t. para-epiglottica*** |
| --- | --- | --- | --- | --- | --- |
| foxes | + | +-++ | ++-+++ | ++ | - |
| raccoon dogs | +-++ | +-++ | ++-+++ | +-++ | - |
| mongooses | -/+ | + | +-++ | - | + |
| raccoons | + | + | ++-+++ | ++ | - |
| dogs | ++ | + | ++ | +-++ | - |
| skunks | -/+ | - | +-++ | nd | - |

t.: *tonsilla*; -: no immune cells or MALT; +: single immune cells or mild MALT; ++: few immune cells or moderate MALT; +++: numerous immune cells or intense MALT; nd: not done..

S**upplementary Table S2.** **Detection of SAD L16 GFP in palatine tonsils by RT-qPCR.** Each of two animals per species and time point (A1 and A2) received 10^8.0^ FFU SAD L16 GFP by direct oral application. Ct‑values ≥ 38: (-) negative

|  | **days post vaccination** | | | | | | | | | |
| --- | --- | --- | --- | --- | --- | --- | --- | --- | --- | --- |
|  | **1** | | **2** | | **3** | | **4** | | **10** | |
|  | A1 | A2 | A1 | A2 | A1 | A2 | A1 | A2 | A1 | A2 |
| foxes | 29.85 | - | 29.61 | - | 29.89 | - | 35.50 | 27.49 | - | 30.49 |
| raccoon dogs | - | - | 33.60 | 22.69 | 29.73 | 31.57 | 34.57 | 31.21 | 29.02 | 36.56 |
| mongooses | 32.42 | 32.40 | 26.29 | 25.63 | 29.23 | 36.90 | 31.71 | 31.59 | 35.86 | - |
| raccoons | - | 34.37 | 35.05 | - | - | - | 35.86 | - | - | - |
| dogs | - | - | - | - | - | 36.97 | 37.17 | - | - | - |
| skunks | 37.70 | 34.43 | 35.60 | - | - | - | - | - | - | - |

**Supplementary Table S3.** **Mean positivity rates of RT-qPCR positive tissue samples.** Animals were grouped in assumed responsive (fox – raccoon dog – mongoose) and refractory (raccoon – dog – skunk) species and the positivity rate were averaged.

|  | fox – raccoon dog – mongoose | | | raccoon – dog – skunk | | |
| --- | --- | --- | --- | --- | --- | --- |
|  | mean | SD | n | mean | SD | n |
| lnn. mandibularis | 16.67 | 4.71 | 3 | 6.67 | 9.43 | 3 |
| lnn. parotideus | 16.67 | 17.00 | 3 | 13.33 | 18.86 | 3 |
| lnn. retropharyng. | 16.67 | 17.00 | 3 | 26.67 | 20.55 | 3 |
| mucosa | 56.67 | 12.47 | 3 | 13.33 | 12.47 | 3 |
| lingual ground | 60.00 | 20.00 | 2 | 23.33 | 4.71 | 3 |
| t. palatina | 76.67 | 12.47 | 3 | 26.67 | 4.71 | 3 |
| t. pharyngea | 46.67 | 18.86 | 3 | 33.33 | 12.47 | 3 |
| soft palate | 50.00 | 10.00 | 2 | 23.33 | 12.47 | 3 |
| tongue | 53.33 | 20.55 | 3 | 6.67 | 9.43 | 3 |

**Supplementary Table S4.** **Detection of SAD L16 GFP in tissue samples of oropharyngeal tract from foxes by RT‑qPCR.** Each of two foxes per time point (Fox1 and Fox2) received 10^8.0^ FFU SAD L16 GFP by direct oral application. Ct-values ≥ 38: (-) negative

|  | **days post vaccination** | | | | | | | | | |
| --- | --- | --- | --- | --- | --- | --- | --- | --- | --- | --- |
|  | **1** | | **2** | | **3** | | **4** | | **10** | |
|  | Fox1 | Fox2 | Fox1 | Fox2 | Fox1 | Fox2 | Fox1 | Fox2 | Fox1 | Fox2 |
| lnn. mandibularis | - | - | - | 37.92 | - | - | 37.37 | - | - | - |
| lnn. parotideus | - | - | - | - | - | - | - | - | - | - |
| lnn. retropharyng. | - | - | - | - | 36.83 | - | - | - | - | - |
| mucosa | 34.94 | - | - | 35.12 | 36.60 | 34.45 | - | - | - | - |
| lingual ground | 34.88 | - | - | 35.84 | - | 32.16 | 35.08 | - | - | - |
| t. palatina | 29.85 | - | 29.61 | - | 29.89 | - | 35.50 | 27.49 | - | 30.49 |
| t. pharyngea | - | - | - | - | - | - | - | 37.67 | - | - |
| soft palate | - | - | - | - | - | 35.41 | 36.76 | - | 37.58 | 35.47 |
| tongue | 35.74 | - | - | - | 36.52 | 34.79 | - | - | - | - |

**Supplementary Table S5.** **Detection of SAD L16 GFP in tissue samples of oropharyngeal tract from raccoon dogs by RT‑qPCR.** Each of two raccoon dogs per time point (RD1 and RD2) received 10^8.0^ FFU SAD L16 GFP by direct oral application. Ct-values ≥ 38: (-) negative

|  | **days post vaccination** | | | | | | | | | |
| --- | --- | --- | --- | --- | --- | --- | --- | --- | --- | --- |
|  | **1** | | **2** | | **3** | | **4** | | **10** | |
|  | RD1 | RD2 | RD1 | RD2 | RD1 | RD2 | RD1 | RD2 | RD1 | RD2 |
| lnn. mandibularis | - | - | 37.21 | - | - | 31.76 | - | - | - | - |
| lnn. parotideus | - | 36.52 | - | 36.86 | - | - | - | - | 34.36 | 37.75 |
| lnn. retropharyng. | - | - | - | 32.33 | 29.12 | 29.95 | - | - | 28.36 | - |
| mucosa | - | - | - | 33.17 | 31.78 | 36.30 | 32.29 | 35.04 | - | 35.89 |
| lingual ground | - | - | 33.66 | 25.82 | 26.09 | 23.44 | 35.48 | 33.22 | 35.46 | 36.00 |
| t. palatina | - | - | 33.60 | 22.69 | 29.73 | 31.57 | 34.57 | 31.21 | 29.02 | 36.56 |
| t. pharyngea | - | - | 33.91 | 32.43 | 33.29 | 31.73 | 34.55 | 29,49 | 37.37 | - |
| soft palate | - | - | - | 34.84 | 29.55 | 32.00 | 33.01 | 33.69 | 37.76 | - |
| tongue | 36.03 | 34.99 | 29.20 | 34.44 | 32.45 | 32.79 | 34.53 | 35.68 | - | - |

**Supplementary Table S6.** **Detection of SAD L16 GFP in tissue samples of oropharyngeal tract from mongooses by RT‑qPCR.** Each of two mongooses per time point (MG1 and MG2) received 10^8.0^ FFU SAD L16 GFP by direct oral application. Ct-values ≥ 38: (-) negative; blank space: not determined

|  | **days post vaccination** | | | | | | | | | |
| --- | --- | --- | --- | --- | --- | --- | --- | --- | --- | --- |
|  | **1** | | **2** | | **3** | | **4** | | **10** | |
|  | MG1 | MG2 | MG1 | MG2 | MG1 | MG2 | MG1 | MG2 | MG1 | MG2 |
| lnn. mandibularis | 37.40 | - | - | - | - | - | - | - | - | - |
| lnn. parotideus | 33.92 | - | - | - | - | - | - | - | - | - |
| lnn. retropharyng. | - | - | - | - | - | - | - | - | - | - |
| mucosa | 33.84 | 35.05 | 34.80 | - | 35.56 | 34.64 | - | - | - | 35.39 |
| lingual ground |  |  |  |  |  |  |  |  |  |  |
| t. palatina | 32.42 | 32.40 | 26.29 | 25.63 | 29.23 | 36.90 | 31.71 | 31.59 | 35.86 | - |
| t. pharyngea | 33.50 | - | 32.09 | 32.73 | 31.23 | - | - | - | - | 35.09 |
| soft palate |  |  |  |  |  |  |  |  |  |  |
| tongue | 33.25 | 35.25 | 33.36 | - | 35.41 | - | - | - | - | - |

**Supplementary Table S7.** **Detection of SAD L16 GFP in tissue samples of oropharyngeal tract from raccoons by RT‑qPCR.** Each of two raccoons per time point (RC1 and RC2) received 10^8.0^ FFU SAD L16 GFP by direct oral application. Ct-values ≥ 38: (-) negative

|  | **days post vaccination** | | | | | | | | | |
| --- | --- | --- | --- | --- | --- | --- | --- | --- | --- | --- |
|  | **1** | | **2** | | **3** | | **4** | | **10** | |
|  | RC1 | RC2 | RC1 | RC2 | RC1 | RC2 | RC1 | RC2 | RC1 | RC2 |
| lnn. mandibularis | - | - | - | - | - | - | - | - | - | - |
| lnn. parotideus | - | - | - | - | - | - | - | - | - | - |
| lnn. retropharyng. | - | 37.76 | - | - | - | - | 37.81 | - | 35.40 | - |
| mucosa | - | - | - | - | - | - | - | - | - | - |
| lingual ground | - | - | - | - | - | - | 36.66 | - | - | 37.78 |
| t. palatina | - | 34.37 | 35.05 | - | - | - | 35.86 | - | - | - |
| t. pharyngea | 35.35 | - | - | 33.10 | 35.27 | 36.47 | 33.16 | 36.40 | - | 37.07 |
| soft palate | - | - | - | - | - | - | - | - | 37.26 | 37.85 |
| tongue | - | - | - | - | - | - | - | - | - | - |

**Supplementary Table S8.** **Detection of SAD L16 GFP in tissue samples of oropharyngeal tract from dogs by RT‑qPCR.** Each of two dogs per time point (Dog1 and Dog2) received 10^8.0^ FFU SAD L16 GFP by direct oral application. Ct-values ≥ 38: (-) negative

|  | **days post vaccination** | | | | | | | | | |
| --- | --- | --- | --- | --- | --- | --- | --- | --- | --- | --- |
|  | **1** | | **2** | | **3** | | **4** | | **10** | |
|  | Dog1 | Dog2 | Dog1 | Dog2 | Dog1 | Dog2 | Dog1 | Dog2 | Dog1 | Dog2 |
| lnn. mandibularis | 35.05 | - | - | - | - | 33.27 | - | - | 36.33 | - |
| lnn. parotideus | - | 36.30 | 21.98 | 36.95 | 36.21 | - | - | - | - | 37.03 |
| lnn. retropharyng. | - | - | - | 36.21 | - | - | 37.02 | 36.14 | 36.51 | 37.93 |
| mucosa | - | - | - | - | - | 36.97 | - | 37.17 | - | - |
| lingual ground | - | - | 29.43 | 37.76 | - | - | - | - | 33.51 | 37.03 |
| t. palatina | - | - | - | - | - | 36.97 | 37.17 | - | - | - |
| t. pharyngea | - | - | 34.68 | - | - | - | - | - | - | 36.36 |
| soft palate | - | - | - | - | - | 37.07 | - | - | 35.92 | 37.08 |
| tongue | - | - | - | - | - | - | - | - | 36.22 | - |

**Supplementary Table S9.** **Detection of SAD L16 GFP in tissue samples of oropharyngeal tract from skunks by RT‑qPCR.** Each of two skunks per time point (SK1 and SK2) received 10^8.0^ FFU SAD L16 GFP by direct oral application. Ct-values ≥ 38: (-) negative

|  | **days post vaccination** | | | | | | | | | |
| --- | --- | --- | --- | --- | --- | --- | --- | --- | --- | --- |
|  | **1** | | **2** | | **3** | | **4** | | **10** | |
|  | SK1 | SK2 | SK1 | SK2 | SK1 | SK2 | SK1 | SK2 | SK1 | SK2 |
| lnn. mandibularis | - | - | - | - | - | - | - | - | - | - |
| lnn. parotideus | - | - | - | - | - | - | - | - | - | - |
| lnn. retropharyng. | - | - | - | - | - | - | - | - | - | - |
| mucosa | 34.83 | - | - | - | - | - | - | - | 33.87 | - |
| lingual ground | - | - | 35.33 | - | - | - | - | 34.33 | - | - |
| t. palatina | 37.70 | 34.43 | 35.60 | - | - | - | - | - | - | - |
| t. pharyngea | - | 34.85 | - | - | - | - | - | 33.15 | - | - |
| soft palate | - | - | - | - | - | - | - | - | - | 35.26 |
| tongue | - | - | - | - | - | - | - | - | - | - |
